# Enriching rare variants using family-specific linkage information

**DOI:** 10.1186/1753-6561-5-S9-S82

**Published:** 2011-11-29

**Authors:** Gang Shi, Jeannette Simino, Dabeeru C Rao

**Affiliations:** 1 Division of Biostatistics, Washington University School of Medicine, 660 South Euclid Avenue, St. Louis, MO 63110, USA

## Abstract

Genome-wide association studies have been successful in identifying common variants for common complex traits in recent years. However, common variants have generally failed to explain substantial proportions of the trait heritabilities. Rare variants, structural variations, and gene-gene and gene-environment interactions, among others, have been suggested as potential sources of the so-called missing heritability. With the advent of exome-wide and whole-genome next-generation sequencing technologies, finding rare variants in functionally important sites (e.g., protein-coding regions) becomes feasible. We investigate the role of linkage information to select families enriched for rare variants using the simulated Genetic Analysis Workshop 17 data. In each replicate of simulated phenotypes Q1 and Q2 on 697 subjects in 8 extended pedigrees, we select one pedigree with the largest family-specific LOD score. Across all 200 replications, we compare the probability that rare causal alleles will be carried in the selected pedigree versus a randomly chosen pedigree. One example of successful enrichment was exhibited for gene *VEGFC*. The causal variant had minor allele frequency of 0.0717% in the simulated unrelated individuals and explained about 0.1% of the phenotypic variance. However, it explained 7.9% of the phenotypic variance in the eight simulated pedigrees and 23.8% in the family that carried the minor allele. The carrier’s family was selected in all 200 replications. Thus our results show that family-specific linkage information is useful for selecting families for sequencing, thus ensuring that rare functional variants are segregating in the sequencing samples.

## Background

Genome-wide association studies (GWAS) have achieved great success in recent years, with about 4,000 single-nucleotide polymorphisms (SNPs) found to be associated with hundreds of common complex traits [1]. However, substantial proportions of their heritabilities have not been accounted for. The so-called missing heritability [2] represents the dark matter of the genetic architecture of complex traits. With the ongoing efforts of many larger mega-consortia, additional genetic variants will likely be discovered through the pooling of statistical evidence from more samples. However, common variants discovered through GWAS have small effect sizes [1]. Finding the missing heritability is one of the most challenging tasks for genetic dissection of complex traits. Rare variants, structural variations, gene-gene interaction, and gene-environment interaction, among others, have been suggested as potential sources to be tapped [2]. Rare variants are likely to have much larger effects [3-5]. Recently, evidence from simulation studies has suggested the presence of synthetic association [6]; that is, rare causal variants may be stochastically associated more often with one allele of the common variants identified through GWAS. Hence rare variants that are poorly correlated with common variants may partly explain the missing heritability. With the advent of exome-wide and whole-genome next-generation sequencing technologies [7], finding rare and functional variants becomes feasible [8].

In this work, we investigate a strategic approach for studying rare variants in families. A real example of our approach has been demonstrated recently [9]. Using the Genetic Analysis Workshop 17 (GAW17) simulated data based on genotype data from the 1,000 Genomes Project [10], we investigate the utility of family-specific linkage information for identifying families that are enriched for rare variants. We had the GAW17 simulation answers [11] when conducting the analyses.

## Methods

We used the simulated family data set provided by GAW17, which consists of 697 subjects in 8 extended pedigrees. The 202 founders were chosen from 697 unrelated individuals participating in the 1000 Genomes Project, and their 495 descendants were simulated. See the GAW17 answers [11] for more details about the family data simulation. In total, 200 replications of simulated traits were available for analysis, including three quantitative traits (Q1, Q2, Q4) and one dichotomous trait (Affected). Our analyses were limited to quantitative phenotypes Q1 and Q2.

We first adjusted the phenotypes Q1 and Q2 for age, sex, and smoking effects and used the standardized residuals in the analysis. We conducted variance component linkage analysis on Q1 and Q2. Family-specific logarithm of odds (LOD) scores were computed using QTL*trends*[12] using the identity-by-descent information provided by GAW17. The QTL*trends* program was developed to conduct variance component linkage analysis with interactions; we used a regular variance component model without interactions for this analysis. We analyzed each of the 200 replications; in each replication, we selected one pedigree that had the largest family-specific LOD score. To evaluate the efficacy of enriching rare causal variants using linkage information, we compared the relative frequency among the 200 replications of selecting the correct family (carrying the causal minor allele) with the probability that a randomly selected family will carry the causal minor allele. To save computational burden, we conducted analyses on only 9 and 13 causal genes used for simulating phenotypes Q1 and Q2, respectively. The variants in gene *FLT4* for Q1 and in genes *INSIG1* and *RARB* for Q2 are monomorphic and thus do not contribute to any phenotypic variation; hence they were deemed noncausal in our evaluations. Analyses on these three genes were actually carried out under the null hypothesis.

We also computed effect sizes of the causal genes in terms of phenotypic variances explained within each family and in the total sample. The effect of each variant for each subject was computed from the number of rare alleles and effect (*β*) provided in the answers, assuming an additive model; the effect of each gene was the sum of all causal variants in it. The percentage of explained phenotypic variance was computed from the average ratio of the variance of the gene effect to the total phenotypic variance from 200 replications.

## Results

In Table 1 we show the counts of rare alleles of causal genes for Q1 and Q2 separately in the eight pedigrees. According to the GAW17 answers, phenotype Q1 was influenced by 39 SNPs in 9 genes and Q2 was affected by 72 SNPs in 13 genes. Because linkage analysis is known to have low genomic resolution and our linkage analyses were conducted at the level of genes (not SNPs), we summarize the numbers of rare alleles by gene. Genes having a single nonmonomorphic causal SNP are indicated with an asterisk in the table. Not all families carry rare alleles of the causal SNPs. For instance, the rare allele of the causal SNP C14S1734 in gene *HIF1A* exists in only one founder of family 5, and the rare allele of SNP C6S2981 in gene *VEGFA* exists only in families 1, 2, and 7. In those families whose founder(s) carry rare alleles, some of the rare alleles were transmitted to descendants and some were not. For example, the founder in family 3 did not transmit a copy of the rare allele of the SNP C1S3181 in gene *ELAVL4* to any descendants. On the other hand, 30 descendants in family 7 inherited the rare allele of the causal SNP C4S4935 in gene *VEGFC* from one common founder. In a sequencing study of rare variants, it is desirable to have families that not only carry the causal rare alleles but also have multiple copies transmitted to the descendants.

**Table 1:** Distribution of causal minor alleles of Q1 and Q2 in the eight simulated pedigrees

Gene	Family								Founder	
	
	1	2	3	4	5	6	7	8	Yes	No
Q1										
* ARNT*	3	2	0	0	6	0	0	0	4	7
ELAVL4*	0	0	1	0	0	0	0	0	1	0
* FLT1*	0	0	5	0	28	16	13	12	28	46
* FLT4*	0	0	0	0	0	0	0	0	0	0
HIF1A*	0	0	0	0	1	0	0	0	1	0
HIF3A*	0	0	0	0	3	0	0	0	1	2
* KDR*	14	37	43	31	6	0	93	0	55	169
VEGFA*	4	20	0	0	0	0	22	0	3	43
VEGFC*	0	0	0	0	0	0	31	0	1	30
Q2										
* BCHE*	4	0	0	0	1	0	2	2	5	4
GCKR*	2	0	0	0	0	0	0	0	1	1
* INSIG1*	0	0	0	0	0	0	0	0	0	0
* LPL*	7	20	2	1	0	1	23	7	15	46
PDGFD*	0	0	0	0	0	0	2	0	1	1
* PLAT*	0	1	2	0	0	3	0	0	4	2
* RARB*	0	0	0	0	0	0	0	0	0	0
* SIRT1*	0	0	3	0	0	4	21	0	4	24
* SREBF1*	16	21	0	5	9	12	8	0	13	58
* VLDLR*	2	0	0	0	11	0	6	0	4	15
* VNN1*	35	60	27	15	24	17	77	0	54	201
* VNN3*	24	17	27	0	21	0	42	42	46	127
* VWF*	0	0	0	2	0	0	0	0	2	0

Asterisks indicate a single nonmonomorphic causal variant in the gene.

We evaluated the utility of linkage information for selecting families that are enriched for rare variants for a sequencing study. We computed family-specific LOD scores for each family and examined whether any rare allele existed in the family with the largest family-specific LOD score. Results from analyzing 200 replications are presented in Table 2. The “Expected” column displays the probability that a randomly drawn family will carry at least one rare allele. Because we randomly selected one family out of the eight, the value in this column is the number of families carrying at least one copy of the rare allele divided by 8. The “Observed” column contains the relative frequency, among the 200 replications, of selecting the correct family (carrying the causal minor allele). This represents the overall hit rates of sampling families with linkage information. Because we selected the family with the largest LOD score in each replication, it is possible that the same family is not always selected in all replications. Because of space limitations, we omit details as to which family was selected in each replication and how often each family was selected. *P*-values comparing the relative frequency of families carrying rare alleles when selected using linkage information versus randomly selecting a family are shown in the last column of Table 2.

**Table 2:** Results of selecting one family using the family-specific LOD score for Q1 and Q2

Gene	LOD	LOD_max_	Expected	Observed	*p*-value
Q1					
* ARNT*	0.07	0.09	0.375	0.390	0.33
ELAVL4*	0.09	0.10	0.125	0.070	0.99
* FLT1*	0.12	0.18	0.625	0.850	2.47 × 10^−11^
* FLT4*	0.24	0.24	0	0	NA
HIF1A*	0.03	0.04	0.125	0.090	0.93
HIF3A*	0.65	0.75	0.125	0.010	1.00
* KDR*	0.71	0.76	0.75	0.965	1.09 × 10^−12^
VEGFA*	3.32	2.40	0.375	1.000	<10^−12^
VEGFC*	3.87	4.44	0.125	1.000	<10^−12^
Q2					
* BCHE*	0.12	0.13	0.5	0.555	0.06
GCKR*	0.08	0.09	0.125	0.100	0.86
* INSIG1*	0.09	0.11	0	0	NA
* LPL*	0.16	0.15	0.875	0.925	0.02
PDGFD*	0.12	0.12	0.125	0.205	3.12 × 10^−4^
* PLAT*	0.23	0.20	0.375	0.395	0.28
* RARB*	0.09	0.11	0	0	NA
* SIRT1*	0.15	0.15	0.375	0.485	6.56 × 10^−4^
* SREBF1*	0.28	0.27	0.75	0.875	2.23 × 10^−5^
* VLDLR*	0.12	0.13	0.375	0.480	1.08 × 10^−3^
* VNN1*	0.28	0.23	0.875	0.785	1.00
* VNN3*	0.28	0.23	0.75	0.795	0.07
* VWF*	0.09	0.10	0.125	0.105	0.80

Asterisks indicate a single nonmonomorphic causal variant in the gene. LOD is the average LOD score of linkage analysis in 200 replications. LOD_max_ is the average maximum family-specific LOD score in 200 replications. “Expected” is the chance of selecting a family carrying rare alleles from random selection; “Observed” is the frequency of selecting a family carrying rare alleles by selecting the family with the LOD_max_ score.

As can be seen from Table 2, for some variants the family-specific LOD score provides valuable information for discriminating families with rare alleles from those without. The best results come from genes *VEGFA* and *VEGFC*, both of which have a single causal SNP and carrier families selected in all 200 replications. Comparing the strategy across different genes and variants, we can see that this method works well when rare alleles not only exist in founders but also are transmitted to multiple descendants. The limited number of rare variant copies in a family where founders are carriers but not descendants contributes little to the phenotypic variance, even though the individual genetic effects may be large. For instance, only one founder from family 4 has rare alleles of the two causal SNPs (C12S181 and C12S211) in gene *VWF*. The frequency of picking this family by the family-specific LOD criterion is 0.105, which is close to the random chance of 0.125. For SNPs with only a few copies of the rare alleles transmitted to descendants (e.g., the causal SNP C19S4831 in *HIF3A*), the results are similar.

LOD scores and maximum family-specific LOD scores (LOD_max_) are also presented in Table 2. The LOD score values were computed from the total sample and averaged over 200 replications; the LOD_max_ values represent the averaged largest family-specific LOD scores, which could come from different families across replications. The best linkage evidence is from genes *VEGFA* and *VEGFC*. Gene *VEGFA* has the causal SNP C6S2981, whose rare alleles are carried in families 1, 2, and 7; the average overall LOD score is 3.32, and the LOD_max_ score is 2.40. For gene *VEGFC*, only family 7 has rare alleles of the causal SNP C4S4935, and its average LOD_max_ score is 4.44, which is larger than the overall LOD score of 3.87.

Not surprisingly, for those rare alleles present only in founders, linkage evidence is sparse. For gene *PLAT*, six copies of rare alleles of causal SNPs are distributed in three families, and the average LOD score is only 0.23. Interestingly, for some genes that have limited linkage evidence, selecting families based on the highest family-specific LOD scores still shows a better chance of enriching rare alleles. For example, 28 copies of the causal minor alleles exist in three families for gene *SIRT1*, which has an average LOD of only 0.15. However, selecting the family with the largest family-specific LOD score has a chance of 0.485 to capture some rare alleles, compared with a random chance of 0.375, and the *p*-value associated with this difference is 6.56 × 10^−4^. In this paper, we focus on examining whether or not the selected family would carry any rare causal allele.

Phenotypic variance explained by each gene is presented in Table 3. It is interesting, though not surprising, to see that some SNPs that are rare in the general population explain large portions of the phenotypic variances in families. SNP C4S4935 in gene *VEGFC* has a minor allele frequency (MAF) of 0.0717% and explains 0.1% of the phenotypic variance in the 697 unrelated individuals. However, it explains 7.9% of the phenotypic variance in the eight pedigrees and 23.8% in family 7, which carries the minor allele. This agrees well with a recent study of adiponectin levels that identified a low-frequency variant with MAF of 1.1% in the general population that explained 17% of the phenotypic variance in families and 63% in carriers’ families [9]. On the other hand, because of smaller effective population size in the simulated families compared with 697 unrelated individuals, some rare variants are monomorphic in the eight simulated pedigrees. For example, there are no minor alleles of rare variants captured by the eight families for genes *FLT4*, *INSIG1*, and *RARB*.

**Table 3:** Percentage of phenotypic variance explained by causal genes of Q1 and Q2 in the eight simulated pedigrees

Gene	Family								Total samples
		
	1	2	3	4	5	6	7	8	
Q1									
* ARNT*	1.3	0.1	0	0	2.0	0	0	0	0.3
ELAVL4*	0	0	0.8	0	0	0	0	0	0.08
* FLT1*	0	0	1.4	0	22.2	11.9	2.3	14.6	5.8
* FLT4*	0	0	0	0	0	0	0	0	0
HIF1A*	0	0	0	0	0.07	0	0	0	0.006
HIF3A*	0	0	0	0	0.4	0	0	0	0.04
* KDR*	0.8	1.6	0.9	1.8	0.5	0	1.6	0	1.2
VEGFA*	7.9	24.6	0	0	0	0	14.6	0	9.0
VEGFC*	0	0	0	0	0	0	23.8	0	7.9
Q2									
* BCHE*	2.4	0	0	0	0.07	0	0.06	0.2	0.3
GCKR*	0.3	0	0	0	0	0	0	0	0.04
* INSIG1*	0	0	0	0	0	0	0	0	0
* LPL*	1.8	4.1	0.6	0.4	0	0.3	4.3	2.2	2.1
PDGFD*	0	0	0	0	0	0	0.5	0	0.1
* PLAT*	0	0.02	0.7	0	0	0.08	0	0	0.1
* RARB*	0	0	0	0	0	0	0	0	0
* SIRT1*	0	0	1.0	0	0	4.2	3.4	0	1.3
* SREBF1*	3.8	2.2	0	1.2	2.1	3.0	1.2	0	1.7
* VLDLR*	0.6	0	0	0	4.6	0	3.1	0	1.2
* VNN1*	1.6	2.8	1.8	1.4	1.7	1.3	2.1	0	1.9
* VNN3*	1.7	1.2	1.9	0	1.6	0	2.1	7.8	2.2
* VWF*	0	0	0	1.7	0	0	0	0	0.2

Asterisks indicate a single nonmonomorphic causal variant in the gene.

## Discussion

We have shown that family-specific LOD scores provide valuable information for selecting families that carry rare causal variants. This method performs well even when the effects of rare variants are not large and the aggregate linkage evidence for the entire sample (a replication in this case) is weak. Although linkage analysis has low power to detect unknown small signals, it seems to work well for selecting samples in order to study a locus known to harbor causal variants, for example, a locus confirmed in GWAS. In a nuclear family in which one of the parents has one copy of a rare allele (both parents having the rare allele would be less likely), half of the offspring are expected to carry this rare allele. Hence rare variants could be common in carriers’ families, which are ideal for evaluating associations with rare variants. On the other hand, because of the rarity of variants, not all founders or families carry the rare alleles; hence choosing families randomly from the general population will not guarantee enrichment of rare variants. Therefore an effective sampling strategy is crucial for enriching rare variants using family data.

Besides *VEGFA* and *VEGFC*, some other genes also showed the efficacy of using family-specific linkage information. For instance, the frequency of selecting a family carrying any rare causal allele in *KDR* is 0.965 compared to the frequency for a randomly chosen family, which is 0.75. As we can see in Table 1, six out of eight families carry at least one copy of a rare allele, and multiple causal SNPs exist in this gene. The increased hit rate for *KDR* reflects the fact that those families that do not carry any rare causal alleles are less likely to have the largest family-specific LOD scores compared with the carriers’ families. This is consistent with our other results. On the other hand, this finding does not represent the typical common disease/rare variant scenario that is being investigated by current exome-wide sequencing studies. In fact, in real studies there is probably no need to enrich causal variants to find this gene because it can easily be detected by GWAS.

As mentioned previously, all causal SNPs in genes *FLT4*, *INSIG1*, and *RARB* are monomorphic in the simulated family data, providing an opportunity for us to examine the method under the null hypothesis. With the nominal distribution of a half-half mixture of a chi-square with 1 degree of freedom and a point mass of 1 at 0, the average LOD score is expected to be 0.11 under the null hypothesis. The LOD score for both *INSIG1* and *RARB* averaged over 200 replications is 0.09, which is close to the expected value. *FLT4* has a much higher average LOD score of 0.24; this number is even larger than the average LOD score for *FLT1*, which has 74 causal minor alleles in five pedigrees and explains 5.8% of the phenotypic variance in the eight pedigrees. According to the GAW17 answers, there were no other causal genes in the proximity of *FLT4*. We were not able to identify the source of the inflated linkage evidence for this gene.

Linkage analysis is able to test the effects of both common and rare variants and hence is immune to the synthetic correlations [6] in GWAS. In addition, family data provide a precious opportunity to examine the quality of the genotype data through examining Mendelian inheritance and conditional allele frequencies. Association analysis based on family data was investigated using many GAW17 groups.

## Conclusions

Families that carry causal rare variants for common complex traits provide an invaluable resource for exome-wide and whole-genome sequencing studies. We evaluated a sampling strategy using family-specific linkage information to select samples that carry the rare alleles. This method works well even when the aggregate linkage evidence is small. Therefore this method can be a powerful approach for follow-up studies of loci identified from previous GWAS or other studies.

## Competing interests

The authors declare that they have no competing interests.

## Authors’ contributions

GS developed the concept, performed the statistical analysis, interpreted the data and drafted the manuscript. JS participated the statistical analysis, interpreted the data and revised the manuscript critically. DCR interpreted the data, revised the manuscript critically and gave final approval for publication. All authors read and approved the final manuscript.
